# Cigarette smoke-induced lung inflammation in COPD mediated via CCR1/JAK/STAT /NF-κB pathway

**DOI:** 10.18632/aging.103180

**Published:** 2020-05-28

**Authors:** Kaishun Zhao, Ran Dong, Yanfang Yu, Chunlin Tu, Ying Li, YuJuan Cui, Lei Bao, Chunhua Ling

**Affiliations:** 1 Department of Respiratory Medicine, The First Affiliated Hospital of Soochow University, Jiangsu 215000, China; 2 Department of Respiratory Medicine, Jiading Central Hospital, Shanghai University of Medicine and Health Sciences, Shanghai 201800, China; 3 Department of Pulmonary and Critical Care Medicine, Tongji Hospital, Tongji University School of Medicine, Shanghai 200065, China

**Keywords:** chronic obstructive pulmonary disease, mouse macrophage cell line, C-C chemokine receptor, airway inflammation, smoke-induced inflammation

## Abstract

Inflammation is an important cause of chronic obstructive pulmonary disease (COPD) and its acute exacerbation. However, the critical role of C-C chemokine receptor (CCR)1 in progression of cigarette smoke-induced chronic inflammation remains unclear. We studied CCR1 expression using immunohistochemistry, immunofluorescence, and real-time polymerase chain reaction (RT-PCR) in COPD patients and controls. Cytokine levels in peripheral blood were measured by enzyme-linked immunosorbent assay (ELISA). In vitro, we investigated Janus kinase/signal transducers and activators of transcription (JAK/STAT)/nuclear factor-κB (NF-κB) signaling in cigarette smoke extract-induced or CCR1 deficiency/overexpressed mouse macrophage cell line MH-S by RT-PCR and western blot, and measured the cytokine levels in the supernatant with ELISA. We found that CCR1 expression was upregulated in COPD patients and there was a negative correlation between CCR1 *mRNA* levels and predicted % forced expiratory volume in 1 min. Inflammatory cytokine levels in the peripheral blood were higher in COPD patients than controls, and these were positively correlated with CCR1 levels. CCR1 was shown to play a critical role in regulating smoke-induced inflammation via JAK/STAT3/NF-κB signaling in vitro. CCR1 may play a critical role in airway inflammation in COPD. Additionally, understanding the molecular mechanism may help develop novel methods for the treatment of COPD.

## INTRODUCTION

Chronic obstructive pulmonary disease (COPD) is characterized by persistent respiratory symptoms and concurrent progressive airflow limitation [1–3]. Patients may experience episodes of exacerbated respiratory symptoms, and the frequency of exacerbations requiring hospitalization increases, resulting in significant social and economic burden and one of the major causes of morbidity and mortality worldwide [4, 5]. The pathogenesis of COPD and exacerbations may be associated with inflammatory cells, including macrophages, neutrophils, and T lymphocytes [6, 7]. These cells are crucial in parenchymal destruction and development of airflow limitation in patients with COPD [8, 9].

Chemokines and their receptors regulate leukocyte adhesion and homing, and these receptors play a critical role in trafficking of leukocytes to sites of injury and inflammation [10]. In fact, the cell surface of T cells, natural killer cells, monocytes, macrophages, lymphocytes, and neutrophils express the C-C chemokine receptor (CCR)1 [11]. Previous studies have shown that elevated blood inflammation cells, chemokine levels, and CCR1 expression are associated with increased risk of exacerbations in patients with COPD [12–16]. However, the critical role of CCR1 in the progression of cigarette smoke-induced chronic inflammation remains unclear. We therefore hypothesize that CCR1 enhances airway inflammation via regulation of Janus kinase/signal transducers and activators of transcription (JAK/STAT)/nuclear factor-κB (NF-κB) signaling.

In this study, we aimed to assess CCR1 expression in peripheral blood and bronchial tissues of patients with COPD and participants who served as controls. Furthermore, we investigated chemokine levels in plasma and correlation with lung function and CCR1 expression. We also aimed to examine the inflammatory responses of MH-S cells that overexpressed or were deficient in CCR1 expression that were treated with cigarette smoke extract (CSE).

## RESULTS

### Immunohistochemistry of CCR1 in bronchial mucosa of patients with COPD and controls

**Figure 1:**
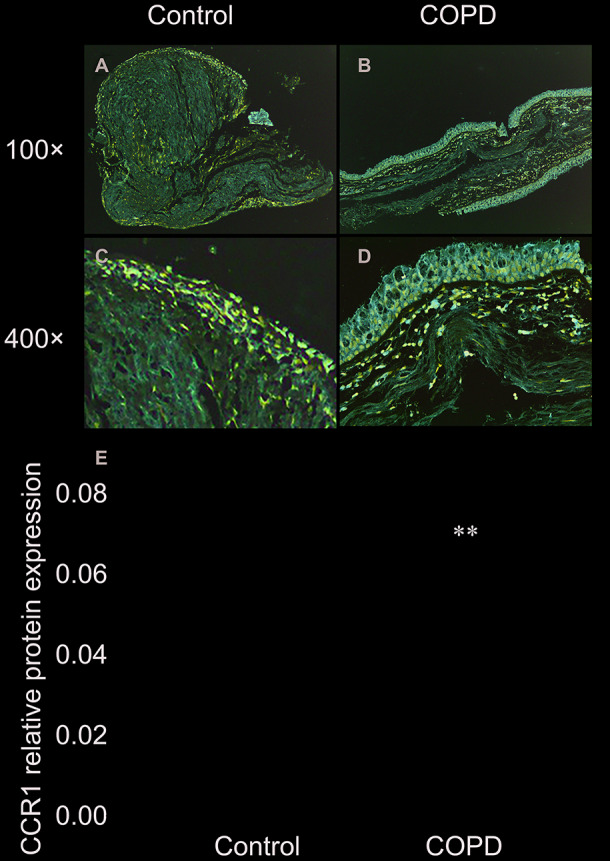
**Immunohistochemistry of CCR1 in the bronchial mucosa of patients with COPD and control.** (**A**) CCR1 expression (brown staining) from a patient with COPD. (**B**) CCR1 expression (brown staining) from a control. (**C**) Representative CCR1 expression (brown staining) from a patient with COPD. (**D**) Representative CCR1 expression (brown staining) from a control. (**E**) Quantification of the histochemistry results, expressed as integral optical density of brown staining in the different views of patients with COPD and controls. The results are presented as mean ± SEM. Original magnification ×200 or ×400. ** p <0.01.

Immunohistochemistry staining showed the presence of CCR1 protein mainly in the airway epithelial cells (Figure 1). We found that expression levels of CCR1 in bronchial mucosa were significantly increased in patients with COPD compared with controls (Figure 1A–1E).

### Immunofluorescence of CCR1 in bronchial mucosa of patients with COPD and controls

**Figure 2:**
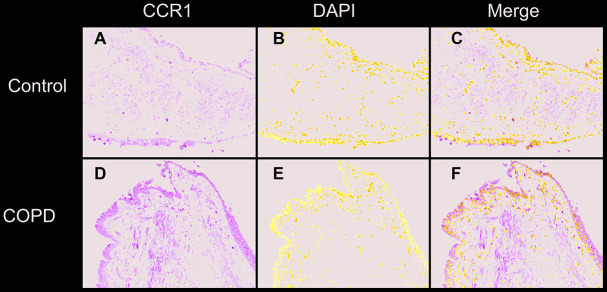
**Immunofluorescence of CCR1 in the bronchial mucosa of patients with COPD and control.** Representative CCR1 expression (green fluorescence) in sections from control (**A**–**C**) and COPD (**D**–**F**).

The CCR1 expression in the trachea was detected by immunofluorescence and confocal microscopy, and was shown as green fluorescence (Figure 2). The ratio of green fluorescence for CCR1 expression was significantly increased in patients with COPD compared with controls. In addition, the enhanced fluorescence was mainly distributed in the mucosa of the trachea (Figure 2). These results were consistent with those illustrated in Figure 1, which indicated that CCR1 expression was significantly increased in patients with COPD.

### The expression of CCR1 mRNA expression in peripheral blood of patients with COPD and controls

**Figure 3:**
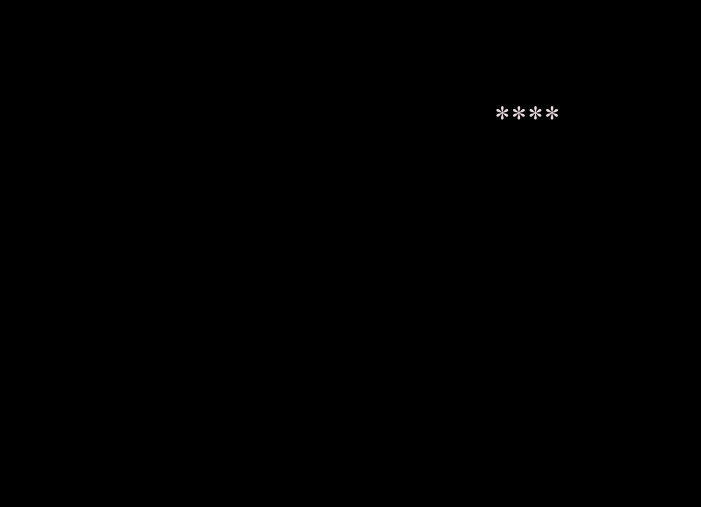
**The expression of CCR1 mRNA in peripheral blood of patients with COPD and control participants.** RT-qPCR detection of CCR1 mRNA expression of peripheral blood. COPD patients show a significantly higher level of CCR1 mRNA compared with the control sample. The results are presented as mean ± SEM (****p <0.0001 vs the control group).

We collected peripheral blood from 35 patients with COPD and 16 controls, then isolated the bone marrow-derived macrophages and analyzed the mRNA level of CCR. Relative mRNA expression of CCR1 compared with the housekeeping gene *Glyceraldehyde-3-Phosphate Dehydrogenase* *(GAPDH)* was significantly higher in macrophages from patients with COPD than controls (Figure 3).

### Cytokine levels in plasma

**Figure 4:**
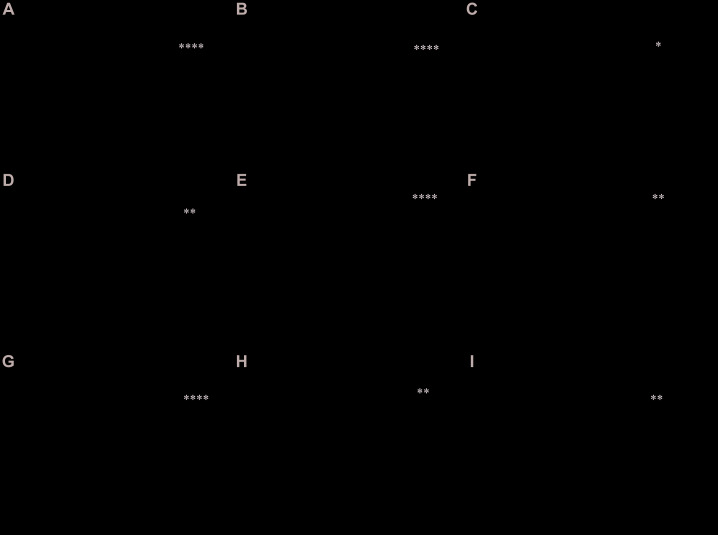
**Cytokine levels in plasma.** ELISA assay of the plasma reveals that COPD patients show a significantly high level of (**A**) IL-6, (**B**) IL-8, (**C**) LIF, (**D**) MCP-1, (**E**) MIP-1 (**F**) α/β, (**G**) RANTES, (**H**) SCF, and (**I**) TNF-α compared with the control group. Data are expressed as mean ± SEM (*p < 0.05, **p <0.01, ****p <0.0001 vs the control group as indicated in the figure).

The IL-8, IL-6, LIF, MCP-1, MIP-1α/β, RANTES, SCF, and TNF-α levels were higher in the plasma of patients with COPD than in controls (Figure 4A–4I). These chemokines indicated persistent airway inflammation in patients with COPD. As a result, we conclude that the elevated level of CCR1 found in the peripheral blood of patients with COPD is consistent with chronic inflammation.

### Relationships between CCR1 mRNA and cytokine expression levels and predicted % forced expiratory volume in 1 min (FEV1%pred)

**Figure 5:**
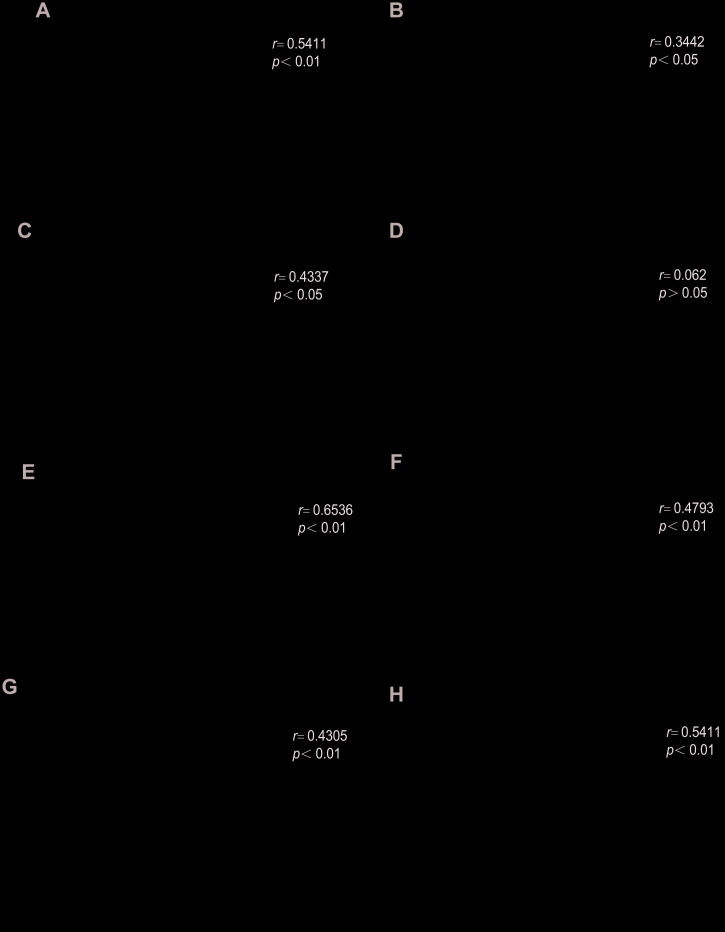
**Relationships between CCR1 mRNA and cytokine expression levels and FEV1%pred.** A negative correlation is observed between CCR1 mRNA levels in the peripheral blood and FEV1%pred in patients with COPD (**A**). There are significant positive correlations between (**B**) CCR1 mRNA levels and IL-8, (**C**) IL-6, (**D**) MIP-1 (**E**) α/β, (**F**) RANTES, (**G**) SCF, and (**H**) TNF-α concentrations.

A negative correlation was observed between CCR1 mRNA levels in the peripheral blood and FEV1%pred in patients with COPD (Figure 5A). Moreover, there were significant positive correlations between CCR1 mRNA levels and IL-8, IL-6, MIP-1α/β, RANTES, SCF, and TNF-α concentration in patients with COPD (Figure 5B–5H).

### Expression of CCR1 and downstream pathways in CSE-induced MH-S cells

**Figure 6:**
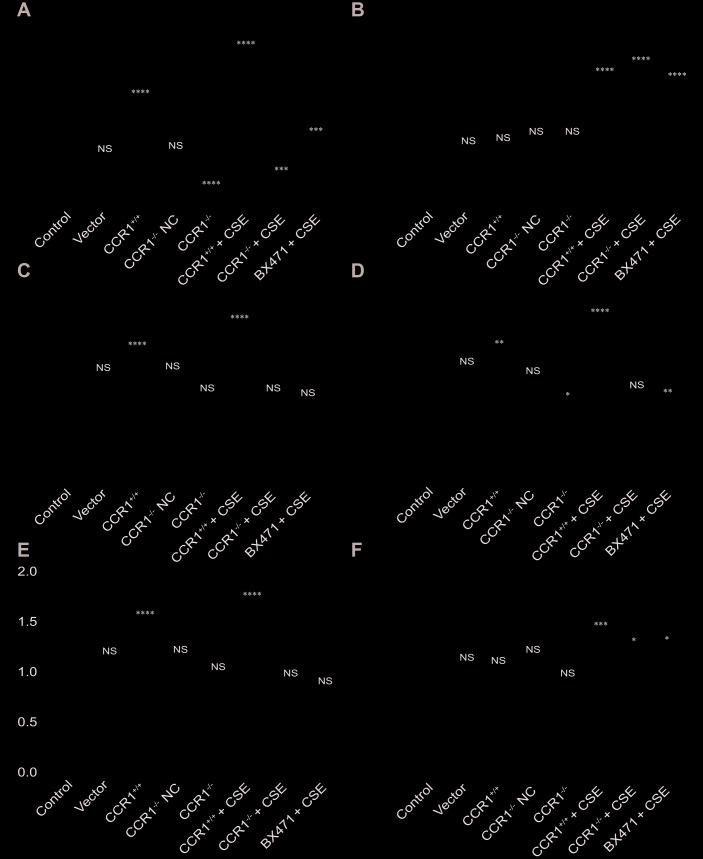
**mRNA expression of CCR1 and downstream pathways in CSE-induced MH-S cells.** (**A**, **C**–**E**) The RT-qPCR results show that once the CCR1 mRNA expression is inhibited, the CCR1/JAK/STAT3/NF-κB mRNA expression decreased significantly in CSE-induced MH-S cells, (**B**, **F**) but not the RANTES and TLR-4 mRNA expression.

**Figure 7:**
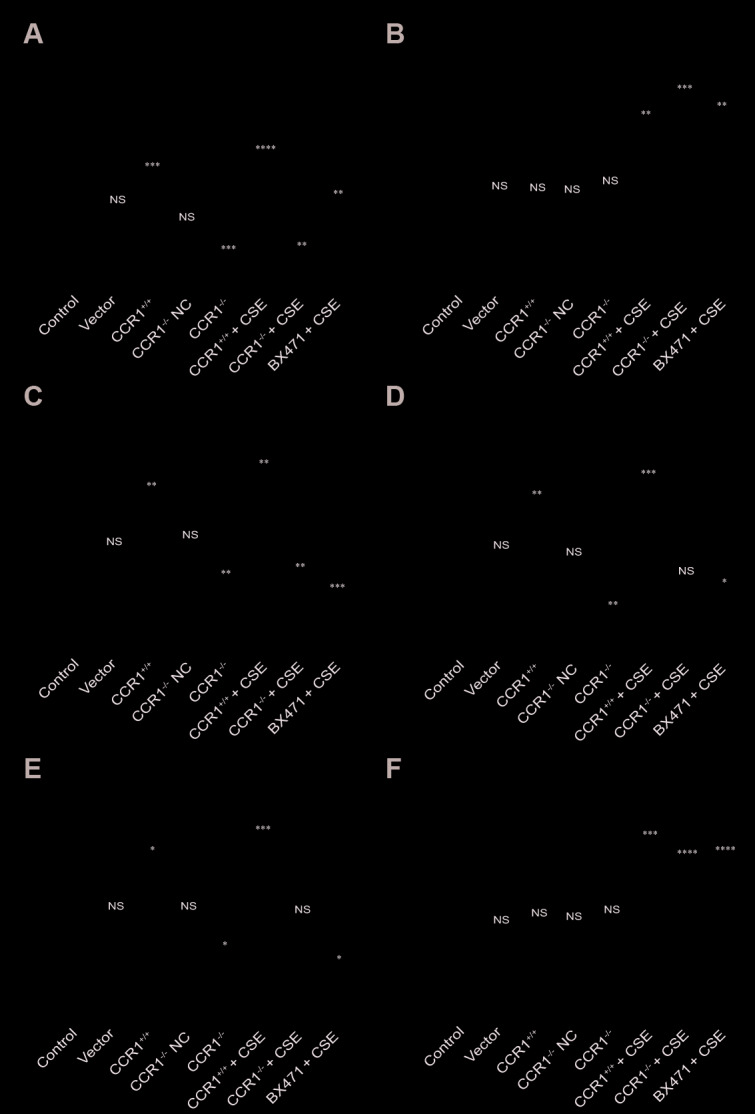
**Protein expression of CCR1 and downstream pathways in CSE-induced MH-S cells.** (**A**, **C**–**E**) The western blot results show that once the CCR1 protein expression is inhibited, the CCR1/JAK/STAT3/NF-κB protein expression decreases significantly in CSE-induced MH-S cells, (**B**, **F**) but not the RANTES and TLR-4.

The RT-qPCR results revealed that once the CCR1 mRNA expression was inhibited, the CCR1/JAK/STAT3/NF-κB mRNA expression decreased significantly in CSE-induced MH-S cells (Figure 6A, 6C–6E), but not the RANTES and toll-like receptor 4 (TLR-4) mRNA expression (Figure 6B, 6F). Similarly, the protein expression detected by western blots were consistent with RT-qPCR results (Figure 7).

### Cytokine secretion in MH-S cellular supernatant

**Figure 8:**
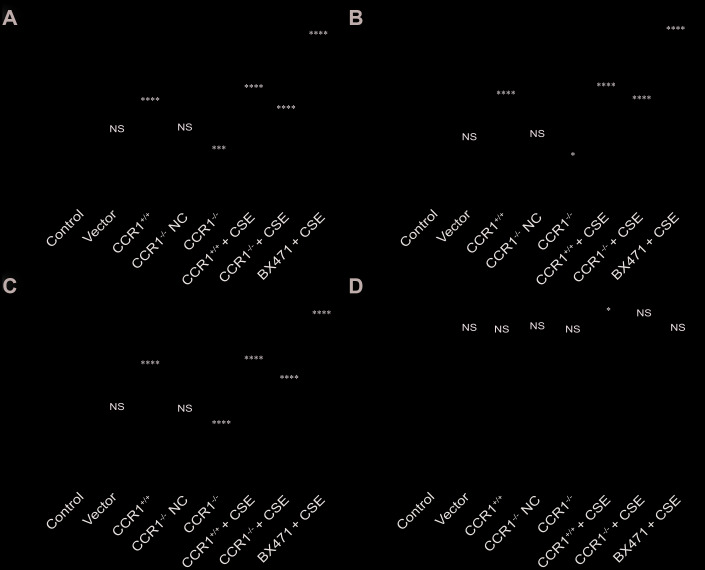
**Cytokine secretion in cellular supernatant.** (**A**–**C**) The CCR1 positive expression may promote the secretion of TNF-α, IL-6, and MIP-1β in cellular supernatant, but these cytokine secretions were also increased in CSE-induced MH-S cells although CCR1 mRNA expression was inhibited. (**D**) However, the concentration of INF-β in cellular supernatant is not correlated with CCR1 positive expression or CSE stimulation.

The CCR1 positive expression may promote the secretion of TNF-α, IL-6, and MIP-1β in cellular supernatant, but these cytokine secretions were also increased in CSE-induced MH-S cells although CCR1 mRNA expression was inhibited (Figure 8A–8C). However, the concentration of INF-β in cellular supernatant is not correlated with CCR1 positive expression or CSE stimulation (Figure 8D).

## DISCUSSION

Our study demonstrated higher expression of CCR1 in patients with COPD, importantly, this study’s finding supports our hypothesis that the critical role of CCR1 in the regulation of smoke-induced inflammation via JAK/STAT3/NF-κB signaling in vitro. We found a negative correlation between CCR1 mRNA levels in the peripheral blood and FEV1%pred, and a positive correlation between CCR1 mRNA levels and chemokine secretions in this cohort of patients with COPD.

Chemokines and their receptors (ChRs) regulate leukocyte adhesion, trafficking and homing. CCR1 belongs to the family of inflammatory ChRs, which are upregulated during inflammation [17]. Notably, CCR1 serves as a receptor for a number of inflammatory chemokines and is upregulated in response to inflammatory stimuli [18–20]. Studies showed that CCR1 was involved in the inflammatory response to cigarette smoking in murine models [21], and it may play a critical role in the pathogenesis of COPD. Similarly, Joubert et al. demonstrated that CCR1 was expressed on human airway smooth muscle cells in patients [22], which may involve in airway remodeling in asthma.

In addition, infection is a significant cause of COPD and its acute exacerbation [23–26]. Acute exacerbations in patients with COPD are often associated with impaired respiratory function for a prolonged period of time and with increased mortality. These prolonged changes may be due to persistent inflammatory changes caused by the infecting pathogen [27, 28]. Patients with COPD are more susceptible to viral infections, and exacerbations are associated with viral infections in up to one-half of COPD cases [29, 30]. Viral infections induce a rapid and potent inflammatory response in different cell types, such as macrophages, fibroblastoid cells, and monocytes that is mediated by an early release of inflammatory cytokines, such as TNF-α, IL-6, and IL-8, and secretion of RANTES. Accumulating evidence shows that ligand binding to CCR1 activates intracellular signaling, leading to cytokine secretion, activation of endocytosis, and clearance of bacteria, environmental particles, and DNA oligonucleotides [10, 31, 32].

Alveolar macrophages (AM)s are believed to play a crucial role in the pathogenesis of COPD and are significantly increased in patients with COPD [33, 34]. AMs release inflammatory mediators, including TNF-α, IL-1β, and IL-6 after CSE stimulation. Alveolar destruction and prolonged lung inflammation occur via these mediators. Additionally, CCR1 is a macrophage scavenger receptor that recognizes and clears potential COPD exacerbating pathogens, such as modified lipids, apoptotic cells, inhaled particles, and microorganisms [35, 36].

In this study, the basal levels of TNF-α and IL-8 were elevated in the peripheral blood specimens of patients with COPD compared with controls. These results are consistent with previous studies that report an increase in systemic and airway cytokines in patients with COPD [37]. However, to the best of our knowledge, we showed that the basal levels of CCR1 were elevated in the bronchial mucosa of patients with COPD.

Further, we previously found that expression of CCR1 increased after CSE exposure in MH-S mouse AMs. Our data implied that chronic cigarette smoke exposure may be associated with high levels of CCR1 found in AMs of patients with COPD, and excessive CCR1 activation in patients with COPD could provoke a dramatic increase in cytokine secretion via JAK/STAT/NF-κB signaling, which is not only effective in facilitating viral clearance but may also further contribute to the exaggerated inflammatory response. However, we found that patients with COPD presented with more severe infections and lung inflammation on exacerbation. We speculated that the reason for this could be due to enhanced CCR1 expression under conditions in which the function of AMs may be deficient in patients with COPD. This scenario could result in an ineffective clearance of viral infections, under conditions in which the inflammatory response is sustained. Clearly, these mechanistic possibilities require further empirical research focused on the involved molecular mechanisms.

Additionally, strong correlations between the CCR1 mRNA levels, proinflammatory cytokine levels, and severity of dampened lung function were observed in patients with COPD. Furthermore, we observed significant negative correlations between the CCR1 mRNA levels and FEV1%pred in patients with COPD, and a significant positive correlation between the CCR1 mRNA expression levels and the IL-8 and TNF-α levels. Overall, these results seem to support the notion that CCR1 could be associated with reduced lung function and permanent stimulation of proinflammatory cytokines that are crucial in COPD progression.

There were some limitations of the study. We utilized a murine macrophages in vitro study; therefore, whether the results can be extrapolated to humans remains unknown. However, our study illustrated the effect of CS-induced lesions on CCR1 expression and the JAK/STAT/NF-κB pathway. in an animal model is required to elucidate the role played by the expression of CCR1 in the progression of COPD.

The results of our study indicate that CCR1 is upregulated in patients with COPD, and that enhanced CCR1 expression may be related to high expression levels of inflammatory cytokines. Importantly, expression of CCR1 expression negatively correlate with FEV_1_%pred, an indication that CCR1 may also play a critical role in the progression of COPD. In addition, this identifies a new target in which novel therapies could be developed to modulate the severity of viral-induced responses in clinical settings. Understanding the molecular mechanism may help develop novel methods for the treatment of COPD.

## MATERIALS AND METHODS

### Chemicals and reagents

All chemicals (except for antibodies and antagonists) were purchased from Sigma-Aldrich (St. Louis, MO, USA). CCR1 antagonist BX417 was purchased from Enzo (Life Sciences, USA). Mouse anti-Rabbit IgG monoclonal antibody (HRP- or FITC- conjugated) and goat anti-mouse IgG monoclonal antibody (HRP-conjugated) were purchased from Abcam (Cambridge, MA, USA). Primary antibodies against CCR1, JAK2, RANTES, STAT3, NF-κ B p65, TLR4, and β-actin were purchased from Cell Signaling (Beverly, MA, USA). Lipofectamine 2000 was purchased from Invitrogen (Carlsbad CA, USA). CCR1 siRNA and siRNA negative control were purchased from Ambion (Life technology, Foster City, CA, USA).

### Participant selection

We obtained endobronchial biopsies (15 patients with COPD and 10 non-COPD control participants) from the Respiratory Ward at Jiading Central Hospital. In addition, peripheral blood was measured in 35 patients with COPD and 16 non-COPD control participants. All the samples were from clinically stable patients, and endobronchial biopsy and peripheral blood for the patients and controls were completely different. The characteristics of the participants are shown in Table 1.

**Table 1:** Participant profile.

**Characteristics**	**Control**	**COPD**	**P-value**
Endobronchial biopsies	N=10	N=12	
Age (year)	64.75±5.8	67.13±3.9	0.166
Sex (male:female)	8:2	11:1	0.571
FEV_1_%predicted	92.44±6.52	41.94±6.12	<0.01
FEV_1_/FVC	89.5±6.02	56.95±3.45	<0.01
Smoking status			
Never	10	1	
Former	0	2	
Current	0	9	
Pack-years	0	37.67±6.37	
Peripheral blood	N=16	N=35	
Age (year)	70.88±6.48	72.61±5.23	0.313
Sex (male:female)	13:3	33:2	0.146
FEV_1_%predicted	81.47±5.73	43.23±5.88	<0.01
FEV_1_/FVC	85.69±4.14	51.68±5.25	<0.01
Smoking status			
Never	16	2	
Former	0	5	
Current	0	28	
Pack-years	0	42.27±3.12	

Data are represented as (mean ± SD) Abbreviations: SD, standard deviation; FEV1, forced expiratory volume in 1 second; FVC, forced vital capacity.

### Immunohistochemical analysis

Immunohistochemistry was conducted to analyze the expression and distribution of CCR1 in bronchial tissues of patients with COPD and controls. Briefly, fixed specimens of the lung tissues (endobronchial biopsies) were embedded in paraffin and sectioned into slices 5 μm in thickness, then dewaxed in xylene and rehydrated. Endogenous peroxidases were inhibited with 0.5% hydrogen peroxide in methanol for 10 min, followed by overnight incubation at 4°C with a rabbit polyclonal IgG antibody against CCR1. Immunodetection was performed with diaminobenzidine, biotinylated goat anti-rabbit IgG reagent, and horseradish peroxidase (HRP) (1:5000, Sigma-Aldrich). After being washed 3 times for 10 min with phosphate buffered saline, these sections were incubated with streptavidin conjugated with HRP at 37°C for 30 min. The sections were incubated in 3, 3’-diaminobenzidine tetrahydrochloride (DAB) for 3 min, then viewed under a light microscope at 400× magnification. The slides were coded and analyzed by an observer without prior knowledge of the experimental procedures. For each lung tissue specimens, a section was randomly chosen and 5 fields were randomly selected from each section. The Image Pro Plus 6.0 system (Media Cybernetics, MD, USA) was used to detect the integral optical density (IOD) of positively stained sections (brown staining). The software measurement of the positively stained area containing CCR1 was used to calculate positive immunostaining (IOD /entire positively stained area). All data from each group were collected at the same time under the same conditions.

### Immunofluorescence analysis

The sections were cut with 4 μm thickness from frozen endobronchial biopsies using a freezing microtome (CM1520; Leica Biosystems, Shanghai, China) and kept at room temperature for 30 min. The sections were then washed with PBS for 5 min three times, incubated for 5-10 min in 3% H_2_O_2_ to eliminate endogenous peroxidase activity, followed by washing with PBS for 5 min twice, and incubated for 1 h with a blocking solution (10% goat serum). Next, the sections were incubated for 30 min with rabbit polyclonal anti-CCR1 antibody, then incubated with FITC-conjugated goat anti-rabbit IgG antibody (1:500; Proteintech, Rosemont, IL, USA) for 30 min at 37°C. Following nuclear staining with DAPI (1:1000; Thermo Fisher Scientific) the sections were observed and analyzed using a fluorescence microscope (Nikon Eclipse TI; Nikon, Tokyo, Japan).

### RNA extraction and real-time PCR

Total RNA from peripheral blood and bronchial tissues were extracted using RNeasy kit (Qiagen, Valencia, CA, USA), and total RNA from MH-S cells was extracted using TRIzol reagent (Thermo Fisher Scientific), according to the manufacturer’s instructions. Single-strand cDNA was synthesized for each sample with oligo (dT) as the primer, using a RevertAid First Strand cDNA Synthesis Kit (Invitrogen) following the manufacturer’s protocol. Total RNA (500 ng) was used in a 7500 Fast Real-Time PCR System (Applied Biosystems, Foster City, CA, USA) with FastStart Universal SYBR Green (Roche, Indianapolis, IN, USA) after cDNA synthesis. The PCR conditions were as follows: initial denaturation at 50°C (2 min) and 95°C (10min), followed by 40 cycles of amplification at 95°C (30 s) and 60°C (30 s). Fold change of gene expression was calculated by the 2^−ΔΔCt^ method relative to the internal reference gene (GAPDH [glyceraldehyde 3-phosphate dehydrogenase]). The sequences of all primers are shown in Table 2. Fold change of the gene expression was calculated by 2^−ΔΔCt^ relative to the internal reference gene (*GAPDH*). The experiments were repeated at least 3 times.

**Table 2:** Primer sequences for real-time PCR.

**Name**	**Primer**	**Sequence**	**Size**
Homo GAPDH	Forward	5‘- TCAAGAAGGTGGTGAAGCAGG -3’	115bp
	Reverse	5‘- TCAAAGGTGGAGGAGTGGGT -3’	
Homo CCR1	Forward	5‘- CAGCCTTCACTTTCCTCACG -3’	170bp
	Reverse	5‘- AACGGACAGCTTTGGATTTCTT -3’	
Mus GAPDH	Forward	5‘- ATGGGTGTGAACCACGAGA -3’	229bp
	Reverse	5‘- CAGGGATGATGTTCTGGGCA -3’	
Mus CCR1	Forward	5‘- AGTGAGAAGAAGGTCAAAGCCG -3’	316bp
	Reverse	5‘- GTTGGTCCACAGAGAGGAAGGG -3’	
Mus NF-kB P65	Forward	5‘- CACCGGATTGAAGAGAAGCG -3’	194bp
	Reverse	5‘- AAGTTGATGGTGCTGAGGGA-3’	
Mus JAK2	Forward	5‘- AGTGGCGGCATGATTTTGTT -3’	181bp
	Reverse	5‘-GCTCGAACGCACTTTGGTAA -3’	
Mus STAT3	Forward	5‘-GACCCGCCAACAAATTAAGA -3’	215bp
	Reverse	5‘- TCGTGGTAAACTGGACACCA -3’	
Mus RANTES	Forward	5‘-TGCTGCTTTGCCTACCTCTC-3’	150bp
	Reverse	5‘-TTGAACCCACTTCTTCTCTG-3’	

### Cell line and CSE prepared

The MH-S mouse AM cell line was obtained from the Cell Collection and Research Center of the Chinese Academy of Sciences. The MH-S cells were propagated in RPMI 1640 (Gibco) supplemented with 10% FBS, 100 U/ml penicillin, and 100 μg/ml streptomycin in a 37 °C 5% CO_2_ incubator. CSE was prepared in a manner similar to that in the previous study [38, 39]. Briefly, rubber tubing connected each cigarette to glass tubing submerged in the media (RPMI 1640) at the bottom of a vacuum filtration flask. Once the cigarette was lit, a vacuum drew cigarette smoke through the RPMI 1640, and deposited soluble components of the smoke into solution. The CSE (100%) was prepared by bubbling smoke from 10 cigarettes in 500 ml of RPMI 1640 at a rate of 0.2 cigarette/min. The pH of the CSE was adjusted to 7.4 and sterile-filtered through a 0.22-μm filter. The CSE was always prepared fresh on the day of the experiment. All the experimental conditions were optimally chosen based on the results of our preliminary experiments.

### Plasmids and siRNA transfection

Plasmids and siRNA transfection in the MH-S cells was performed using Lipofectamine^TM^ 2000 (Invitrogen; Thermo Fisher Scientific, Inc.), according to the manufacturer's protocol. In brief, 10 ul siRNA (20 uM) or 4 ug plasmids were diluted in 100 ul opti-MEM (Gibco), and 5 ul Lipofectamine^TM^ 2000 was diluted in 100 ul opti-MEM, then mixed to make a 200 ul transfection diluent. The transfection diluent was added to the MH-S cells following 24 h cell adaptation on 6-well plates (5x10^5^ cells/well), in a 37 °C 5% CO_2_ incubator. The siRNA sequence was 5'-GCAGCAUAGGAGGCUUCAATTUUGAAGCCUCCUAUGCUGCTT-3'.

### Cytokine enzyme-linked immunosorbent assays

Cytokine concentrations in cell culture supernatant and serum were determined using DuoSet ELISA kits (R&D Systems, Minneapolis, MN, USA), according to the manufacturer’s instructions. In brief, the standard was diluted; then the samples, standards and blank were added to the wells of the plate and incubated for 1 h at 37 °C. The liquid was discarded; the plate was washed 5 times and patted dry. Chromogenic reaction reagent was added and incubated in the dark for 15 min at 37 °C. Finally, stop solution was added, and the absorbance at 450 nm was measured within 10 min. Each experiment was performed in triplicate.

### Western blot

All samples were lysed in 50 μL of lysis buffer (10 mM HEPES, 10 mM KCl, 0.1 mM EDTA, 0.1 mM EGTA, 0.5% NP-40, 1 mM DTT, and protease inhibitors). The lysates were incubated on ice for 30 min with vortexing every 5 min and then centrifuged at 12, 000 g for 15 min at 4°C. The supernatant was collected as protein samples. Protein concentrations (20 μL) were measured using a bicinchoninic acid assay kit (Thermo Fisher) according to the manufacturer’s protocol. Equal amounts of protein (30 μg) were subjected to 10% sodium dodecyl sulfate–polyacrylamide gel electrophoresis. Gels were run at 80 V for 30 min, followed by 120 V for 1 h, before being transferred to a polyvinylidene fluoride membrane (Millipore, Burlington, MA, USA). After blocking with PBS containing 5% nonfat milk for 2 h at room temperature (25°C), the product was incubated overnight at 4°C either with antibodies (diluted 1:1000) against CCR1, JAK2, RANTES, STAT3, NF-κ B p65, TLR4 or β-actin. The membrane was washed 3 times for 5 min with 15 mL of Tris-buffered saline and Tween 20, and then incubated with horseradish peroxidase-conjugated goat anti-rabbit IgG antibody (1:2000; Sigma, Welwyn Garden City, UK) for 1 h at room temperature (25°C). After washing, 1 mL of a chemiluminescent substrate (Thermo Fisher) was added to the membrane. The signal was detected and quantified with an enhanced chemiluminescence system (Image Quant LAS-4000 MINI; GE Healthcare Bio-Sciences, Pittsburgh, PA, USA). The signals specific for proteins in the same lane on the gel were analyzed, which were normalized to β-actin.

### Statistical analysis

Results are presented as mean ± standard error of the mean (SEM), if not stated otherwise. A two-tailed *t*-test was performed for comparison of baseline characteristics and a one-way analysis of variance was used for multiple-comparison statistical analysis, followed by post hoc analysis of the Student–Newman–Keuls q test between pairs of groups. A linear regression was adopted using Spearman’s rank correlation test. Statistical analysis was performed as described in each figure legend, using the GraphPad Prism 7.0 software (GraphPad Software, CA, USA), and a p-value of < 0.05 was considered statistically significant.
